# Combining xQTL and genome-wide association studies from ethnically diverse populations improves druggable gene discovery

**DOI:** 10.21203/rs.3.rs-6700169/v1

**Published:** 2025-05-28

**Authors:** Noah Lorincz-Comi, Wenqiang Song, Xin Chen, Isabela Rivera Paz, Yuan Hou, Yadi Zhou, Jielin Xu, William Martin, John Barnard, Andrew A. Pieper, Jonathan L Haines, Mina Chung, Feixiong Cheng

**Affiliations:** 1 Cleveland Clinic Genome Center, Lerner Research Institute, Cleveland Clinic, Cleveland, OH 44195, USA; 2 Genomic Medicine Institute, Lerner Research Institute, Cleveland Clinic, Cleveland, OH 44195, USA; 3 Department of Quantitative Health Sciences, Lerner Research Institute, Cleveland Clinic, Cleveland, OH 44195, USA; 4 Department of Psychiatry, Case Western Reserve University, Cleveland, OH 44106, USA; 5 Brain Health Medicines Center, Harrington Discovery Institute, University Hospitals Cleveland Medical Center, Cleveland, OH 44106, USA; 6 Geriatric Psychiatry, GRECC, Louis Stokes Cleveland VA Medical Center; Cleveland, OH 44106, USA; 7 Institute for Transformative Molecular Medicine, School of Medicine, Case Western Reserve University, Cleveland 44106, OH, USA; 8 Department of Pathology, Case Western Reserve University, School of Medicine, Cleveland, OH 44106, USA; 9 Department of Neurosciences, Case Western Reserve University, School of Medicine, Cleveland, OH 44106, USA; 10 Department of Population & Quantitative Health Sciences, Cleveland Institute for Computational Biology, Case Western Reserve University School of Medicine, Cleveland, Ohio, USA; 11 Department of Cardiovascular Medicine, Heart, Vascular & Thoracic Institute, Cleveland Clinic, Cleveland, OH 44195, USA; 12 Department of Cardiovascular and Metabolic Sciences, Lerner Research Institute, Cleveland Clinic, Cleveland, OH 44195, USA; 13 Department of Molecular Medicine, Cleveland Clinic Lerner College of Medicine, Case Western Reserve University, Cleveland, OH 44195, USA

**Keywords:** Druggable gene, genome-wide association studies (GWAS), gene-based testing, neurodegenerative disease, expression quantitative trait loci (eQTL)

## Abstract

Repurposing existing medicines to target disease-associated genes represents a promising strategy for developing new treatments for complex diseases. However, progress has been hindered by a lack of viable candidate drug targets identified through genome-wide association studies (GWAS). Gene-based association tests provide a more powerful alternative to traditional single nucleotide polymorphism (SNP)-based methods, yet current approaches often fail to leverage shared heritability across populations and to effectively integrate functional genomic data. To address these challenges, we developed GenT and its various extensions, comprising a framework of gene-based tests utilizing summary-level GWAS data. Using GenT, we identified 16, 15, 35, and 83 druggable genes linked to Alzheimer’s disease (AD), amyotrophic lateral sclerosis, major depression, and schizophrenia, respectively. Additionally, our multi-ancestry gene-based test (MuGenT) uncovered 28 druggable genes associated with type 2 diabetes that previous trans-ancestry or ancestry-specific GWAS had missed. By integrating brain expression and protein quantitative trait loci (e/pQTLs) into our analysis, we identified 43 druggable genes (e.g., *RIPK2, NTRK1, RIOK1*) associated with AD that had supporting xQTL evidence. Notably, experimental assays demonstrated that the *NTRK1* protein inhibitor GW441756 significantly reduced tau hyper-phosphorylation (including p-tau181 and p-tau217) in AD patient-derived iPSC neurons, thus providing mechanistic support for our predictions. Overall, our findings underscore the power of gene-based association testing as a strategic tool for informed drug target discovery and validation based on human genetic and genomic data for complex diseases.

## Introduction

For decades, genetic epidemiologists have endeavored to identify disease-associated genes using findings derived from human genome sequencing (Claussnitzer et al., 2020). The most common approach to discovering these genes has been through genome-wide association studies (GWAS), which estimate marginal associations between millions of single nucleotide polymorphisms (SNPs) and various phenotypes (Uffelmann et al., 2021). Typically, gene-level inferences are drawn by assigning the SNP with the smallest association p-value in a locus (known as the lead SNP) to its nearest physical gene (Tam et al., 2018). However, functional genomic studies indicate that only one-third of lead SNPs accurately tag causal genes (Gusev et al., 2016; Zhu et al., 2016), and only about 5% of lead SNPs are likely to be causal (Farh et al., 2015). Consequently, this conventional methodology necessitates statistical correction for all genome-wide tested SNPs, rather than just the genes to which they are presumed related. This strict correction imposes limitations on statistical power, undermining the ability to detect disease-associated genes that interact with available drugs, referred to as ‘druggable genes’ (Reay & Cairns, 2021; Lago & Bahn, 2022; Qi et al., 2023).

A more powerful approach for generating clinically relevant insights into biology and drug discovery is gene-based association testing (Huang et al., 2011). Gene-based association testing performs a joint test on a gene-specific SNP set. While existing gene-based association tests using summary GWAS data represent efficient screening tools, each standard method relies on a statistic with an unknown null distribution. Accurately estimating this distribution is critical for reducing false discoveries of disease-causing druggable genes. Past efforts to address this issue have involved numerical approximations (i.e. Pascal; Lamparter et al., 2016; MAGMA, de Leeuw et al., 2015), transformation based on linkage disequilibrium (LD) structure that is seldom precisely known (DOT; Vsevolozhskaya et al., 2020), simulation (Pascal; VEGAS; Liu et al., 2010), or permutations (MAGMA, LDAK; Berrandou et al., 2023). However, these various methods often lack strong theoretical grounding and can inflate both false positive and negative rates (Zhang et al., 2020; Hof & Speed, 2024; Berrandou et al., 2023; Hecker et al., 2017; Yurko et al., 2020). Other gene-based inference methods attempt to assess causality using summary data by integrating GWAS results with transcriptomic information (e.g., S-PrediXcan, TWAS, SMR, EMAGMA, cis-MR; Barbeira et al., 2018, Gusev et al., 2016, Zhu et al., 2016, Gerring et al., 2019, Gkatzionis et al., 2023), but these approaches rely on additional assumptions that may be difficult to verify and are not universally applicable.

The principal strength of gene-based methods is their capacity to perform a single test per gene, typically requiring only GWAS summary statistics and an LD reference panel. Nevertheless, these methods are limited by their inability to integrate heterogeneous GWAS and LD information across different ancestral populations, to accommodate diverse transcriptomic data types such as gene expression and protein abundance, and to prioritize driver genes in a gene-dense locus. Given that many complex diseases emerge from multidimensional risk factors within diverse biological contexts, there is an urgent need to harness functional genomic data for efficiently screening disease-associated drug targets.

In this study, we present a statistical framework, GenT, designed to test the gene-based null hypothesis with greater theoretical support. This framework states the exact null distribution of the gene-based test statistic when LD is known, and a highly precise null distribution when it is estimated. We leverage these derived properties to extend standard gene-based association testing to integrate multi-ancestry data (MuGenT), test for the presence of a shared disease-xQTL (molecular quantitative trait locus) signal, and finemap multiple genes in a locus using their test statistics to identify putative driver genes. We additionally propose three adaptations of the multi-ancestry test to better understand gene-disease association heterogeneity across ancestral populations. We applied these methods to identify candidate druggable genes associated with several complex diseases, including Alzheimer’s disease (AD), major depression (MDD), schizophrenia (SCZ), amyotrophic lateral sclerosis (ALS), and type 2 diabetes (T2D). Our analyses uncovered multiple potential druggable genes for each disease, supported by empirical evidence from experimental and clinical studies, providing valuable insights to guide future mechanistic investigations and drug discovery efforts.

## Results

**Figure 1:**
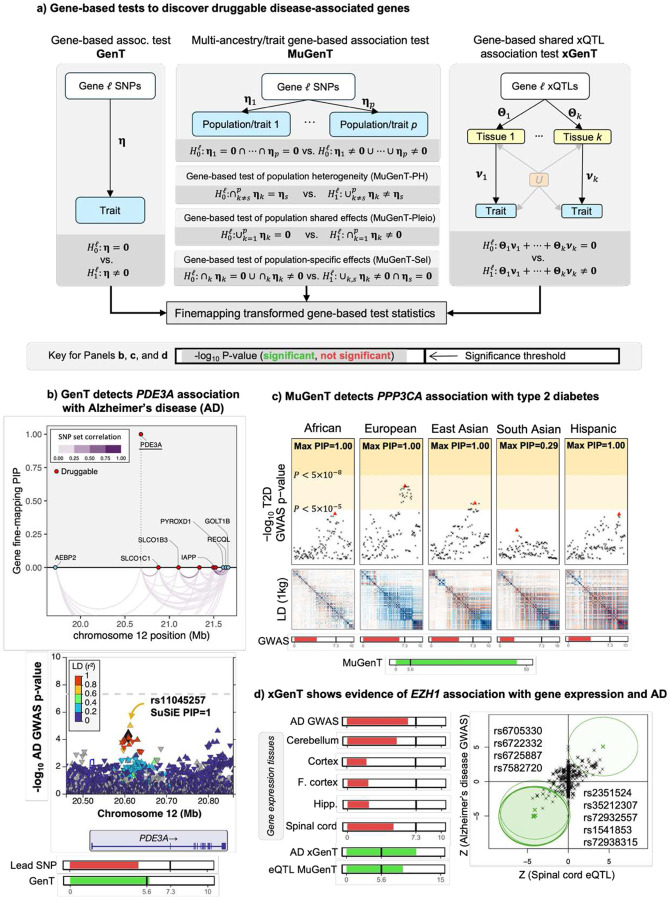
Overview of methods (a) visual descriptions of the gene-based association tests that we introduce and their corresponding null hypotheses. We introduce GenT, MuGenT, xGenT, and three follow-up multi-ancestry tests: MuGenT-PH, MuGenT-Pleio, MuGenT-Sel. (b) Motivating example of GenT which detects a novel druggable gene associated with Alzheimer’s disease (AD) that has supporting gnee- and SNP-level fine-mapping evidence. (c) Motivating example of MuGenT which detects a novel druggable gene associated with T2D by leveraging genetic similarity across populations. (d) Motivating example of xGenT which detects a novel association between *EZH1* and AD risk with colocalized evidence from cis expression quantitative loci (eQTL) from five brain tissues. In the far-right panel, annotated SNPs are jointly significant in the AD GWAS and spinal cord eQTL studies and their 95% confidence ellipses are displayed around their Z-statistics of marginal association.

Figure 1 summarizes our comprehensive set of marginal gene-based association tests and illustrates their application to real data through our single-trait association (GenT; Figure 1b), multi-trait/ancestry association (MuGenT; Figure 1c), and xQTL-integrated association (xGenT; Figure 1d) tests. Gene-based test statistics from all methods are asymptotically eligible for fine-mapping under a simple transformation to identify putative driver genes in a locus (*cf.* Methods). The GenT test employs the standard gene-based association method using summary-level GWAS data, which calculates the sum of correlated SNP chi-square statistics for SNPs in/around a given gene body. Here we introduce a new formulation of its null distribution, which we demonstrate in the **Supplement** to be exact when the LD structure is known and extremely precise when estimated across a range of conditions.

MuGenT expands upon GenT to accommodate multiple ancestral populations, harnessing their shared heritability and genetic correlation to enhance the power of the joint testing. This approach can also be applied to multiple phenotypes measured within the same population. xGenT extends GenT by incorporating xQTL effect sizes from diverse sources as weights, enabling an inference of shared disease and xQTL signals in a gene-specific SNP set. Each of these methods (GenT, MuGenT, and xGenT) leverages polygenicity and requires a less stringent corrected significance threshold than conventional SNP-based gene inference from GWAS. This relaxation increases the power of gene-based association testing to identify druggable genes associated with disease. GenT tests the null hypothesis that no SNP in the gene set is associated with the trait. MuGenT applied to a single trait measured in multiple ancestry groups tests the null hypothesis that no SNP in the gene set are associated with the trait in any group. xGenT tests the null hypothesis that no SNP is simultaneously associated with the trait and any xQTL trait which is modelled.

**Figure 2:**
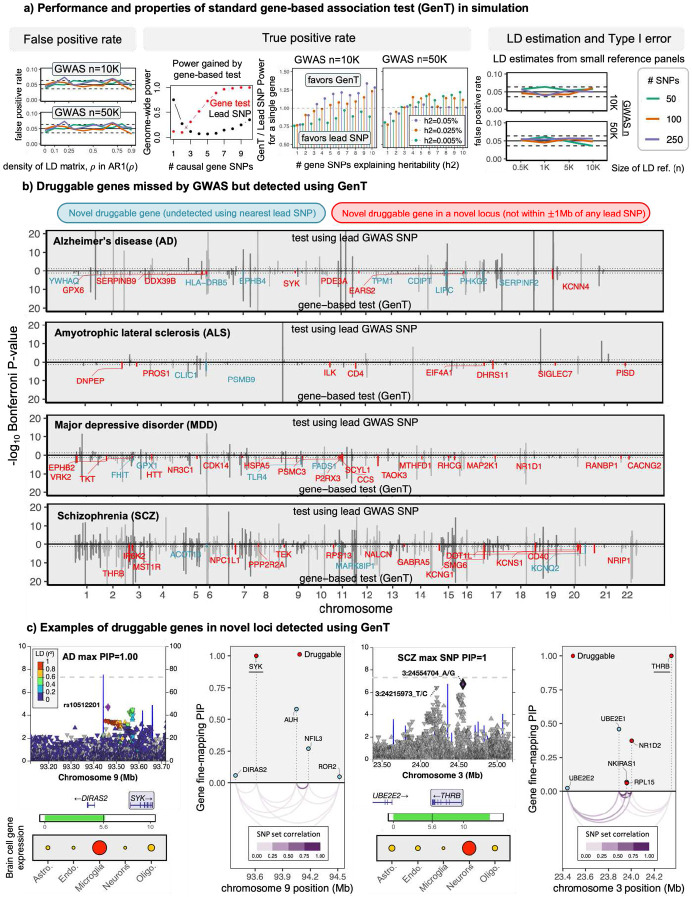
Gene-based association testing for four phenotypes (a) results of simulations performed to evaluate the statistical properties of MuGenT. (b) Manhattan plots of gene-based test (GenT) p-values for four neuropsychiatric traits. Annotated genes are druggable and undetected in the respective GWAS. (c) Examples of druggable genes detected in novel loci for AD and SCZ using GenT and posterior inclusion probabilities from gene- and SNP-level fine-mapping. Displayed under SNP-level locus plots are cell-specific gene expression proportions in human cortex tissue from Zhang et al. (2016). ‘Astro.’: fetal and mature astrocytes; ‘Endo.’: endothelial cells; ‘Oligo.’: Oligodendrocytes. Larger circles and darker red colors indicate greater FPKM values.

The results presented in Figure 2 highlight examples of druggable genes detected exclusively through gene-based association testing, and not through traditional GWAS, for AD and T2D. These genes represent new candidate drug targets previously not detected in lead SNP analyses. For example, *PDE3A* exhibited a GenT false discovery rate (FDR) q-value of 7.6x10^−5^, while the smallest FDR q-value for any SNP in this locus (±1Mb) from AD GWAS was 0.148. Gene-level fine-mapping via SuSiE (Zou et al., 2022) of genes in this locus prioritized only *SYK* (PIP=1.00), and SNP-level fine-mapping of SNPs ±100Kb of *SYK* suggests that rs11045257 is likely causal (PIP=1.00). Similarly, the *PPP3CA* gene did not achieve genome-wide significance in any ancestry-specific GWAS for T2D, whether from European (EUR), African (AFR), East or South Asian (EAS, SAS), or Hispanic (HIS) populations, but its MuGenT FDR q-value was 4.9x10^−45^. Gene-level fine-mapping in the *PPP3CA* locus prioritized *PPP3CA* and *BANK1* (PIPs=1.00), and SNP-level fine-mapping supports the likelihood of causal T2D SNPs in the *PPP3CA* locus ±100Kb across all populations except for SAS.

**Figure 3:**
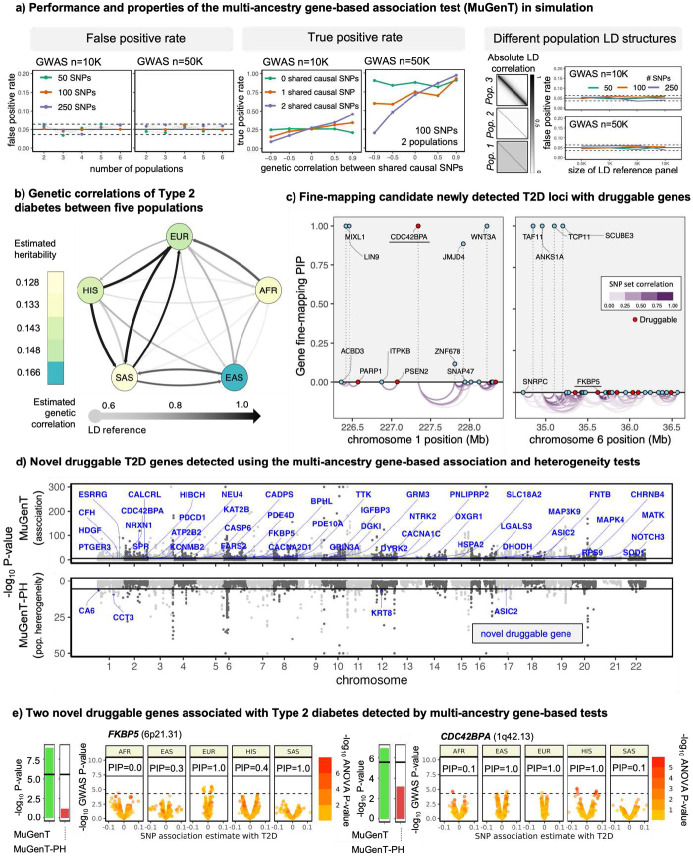
Multi-ancestry gene-based association test applied to T2D (a) results of simulations performed to evaluate the statistical properties of MuGenT. (b) Estimated genetic correlations of T2D between populations. Populations at arrow tails indicate the population which was used as the LD reference when calculating genetic correlation with the trait at the arrowhead. LD reference panels were calculated using 1000 Genomes Phase 3. (c) Gene-level fine-mapping in two loci detecting novel druggable genes with GenT. These results provide supporting evidence for the *CDC42BPA* association with T2D but not *FKBP5.* (e) Volcano plots of ancestry-specific T2D GWAS p-values, MuGenT and MuGenT-PH p-values, and SuSiE fine-mapping posterior inclusion probabilities for two example druggable genes in novel T2D loci.

**Figure 4:**
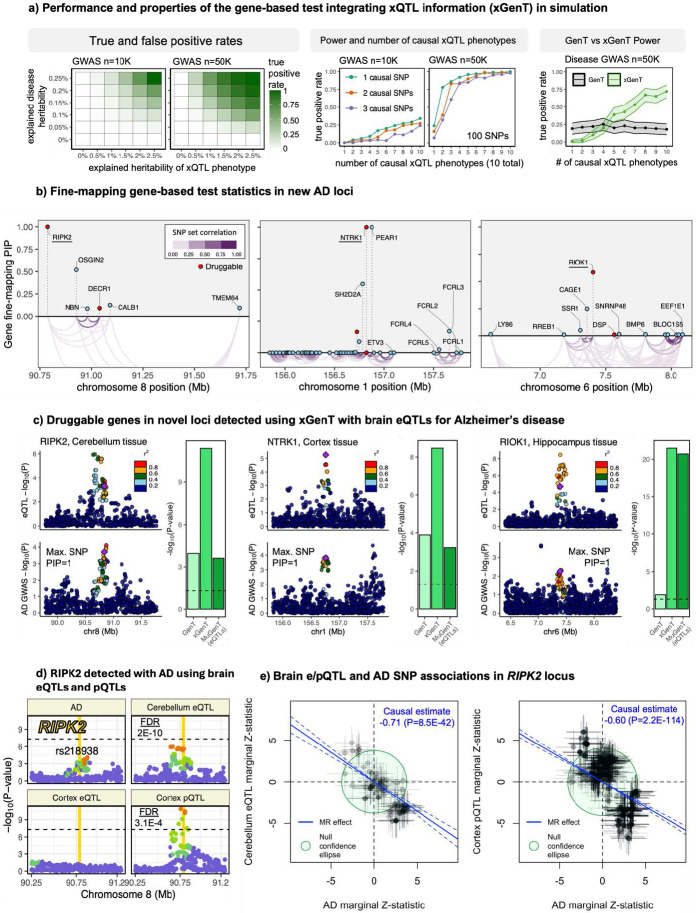
Gene-based tests of Alzheimer’s disease integrating eQTLs (a) results of simulations performed to evaluate the statistical properties of xGenT. (b) Gene-level fine-mapping results for three novel druggable genes detected using xGenT with AD. (c) Locus-specific association plots for three examples of novel druggable genes and the gene expression tissues with corresponding evidence. (d) SNP-level locus plots for *RIPK2* illustrating potential xQTL tissue and functional specificity. Warmer colors indicate stronger LD with the lead SNP; cooler colors indicate lower LD. The yellow region represents the hg19 start and end positions of *RIPK2* and the horizontal dashed line indicates the level of genome-wide significance. (e) Scatterplots of SNP-specific marginal Z-statistics measuring association with AD and cerebellum eQTLs (left) and cortex pQTLs (right) in the *RIPK2* locus. The blue line is the Mendelian Randomization causal estimate using MRBEE.

Figure 1d illustrates that xGenT, integrating eQTLs from five brain tissues, can detect an association between *EZH1* expression and AD that GWAS alone fails to identify. Additionally, it demonstrates that MuGenT can simultaneously test multiple xQTL types within the same population. Simulation results showcased in Figures 2a, 3a, and 4a suggest that our gene-based association tests maintain controlled false positive rates, despite variations in GWAS sample sizes, SNP heritability, LD structure or estimation accuracy, and the number of tested SNPs. These tests generally exhibit greater power than gene-based inference from lead SNPs, benefitting not only from reduced multiple testing but also from leveraged polygenicity, while lead SNP-based methods lose power in such scenarios (Figure 2a). **Supplement Sections S2-5,13** provide additional evaluations of Type I error for GenT, MuGenT and its post-hoc tests, and xGenT, demonstrating that each test has well-controlled false positive rates.

A compelling case study highlighting the advantages of GenT is the association between *SYK* and AD (Figure 2c). The smallest AD GWAS P-value within 50kb of the *SYK* gene body was 2.1x10^−5^ (rs10512201), which falls short of genome-wide significance. However, gene-level fine-mapping in the *SYK* locus (1Mb including *DIRAS2, SYK, AUH, NFIL3,* and *ROR2*) prioritized only *SYK* (PIP=1.00), and SNP-level fine-mapping identified seven SNPs within 100Kb of *SYK* that had PIPs greater than 0.95. As the number of causal SNPs at the *SYK* locus increases, traditional gene-based inference using the lead SNP loses power. By contrast, gene-based association testing using GenT with 1,492 proximal SNPs substantially enhanced the power to detect the *SYK* association with AD (FDR q=8.0x10^−5^; Figure 2a). Remarkably, it is approximated that the AD GWAS would have needed to recruit approximately 670K additional participants to achieve genome-wide significance at 90% power to detect the *SYK* association using the lead SNP rs10512201.

### GenT discovers potential druggable targets

We employed GenT to analyze 18,257 genes across four complex diseases: Alzheimer’s disease (AD), amyotrophic lateral sclerosis (ALS), major depressive disorder (MDD), and schizophrenia (SCZ), with results presented in Figure 2b-c. All analyses utilized GWAS summary statistics from predominantly European (EUR) populations and EUR LD reference panels from the 1000 Genomes Phase 3 (1000 Genomes Project Consortium, 2015). We identified 89 druggable genes associated with AD, 28 druggable genes associated with ALS, 65 druggable genes associated with MDD, and 258 druggable genes associated with SCZ, using a Bonferroni-corrected 5% significance threshold. Of these, 49 were finemapped (PIP>0.9) in their loci for AD, 23 for ALS, 58 for MDD, and 177 for SCZ.

Notably, within the set of associated genes, we identified 75 druggable genes for AD, 26 druggable genes for ALS, 58 druggable genes for MDD, and 218 druggable for SCZ that were undetectable by the conventional lead SNP method (Figure 2b). We also found 16, 15, 35, and 83 genes linked to AD, ALS, MDD, and SCZ, respectively, that reside beyond 1Mb from any previously identified GWAS genes for each disease, spanning 10, 12, 24, and 57 independent loci (>1Mb separation). These genes are located within loci not captured by standard GWAS and encode druggable proteins. Examples of genes with functional studies supporting their disease association, yet not meeting the threshold for genome-wide significance in GWAS, include *KCNN4* in AD (Kosoy et al., 2022), *PISD* in ALS (Phan et al., 2023), *EPHB2* in MDD (Zhen et al., 2018), and *KCNQ2* in SCZ (Choi et al., 2018).

Two examples of druggable genes with supporting gene- and SNP-level fine-mapping evidence are displayed in Figure 2c and demonstrate that disease-associated druggable genes can still be prioritized as putative local signal drivers even when GWAS evidence falls short of genome-wide significance. We found at least one overlapping druggable gene for each pair of diseases, for example: *EPHX2* for AD and SCZ, *PPP3CA* and *CD40* for MDD and SCZ, and *ATP6V1G2, PRKD1,* and *PSEN2* for ALS and SCZ. Comprehensive GenT fine-mapping results for all tested genes are available in Supplementary Tables 1-4 for AD, ALS, MDD, and SCZ, along with their SNP-level SuSiE fine-mapping results in Supplementary Tables 8-11 and gene-level fine-mapping results in Supplementary Tables S19-S23. In total, we applied gene-based association testing using GenT to 38 complex traits, and these results are publicly accessible at the repositories listed in the Data Availability section.

### Multi-ancestry/trait gene-based association tests (MuGenT) identifies druggable targets across diverse populations

We applied our multi-ancestry/trait gene-based association test (MuGenT) to analyze five ancestral populations to identify druggable genes associated with T2D. The results are displayed in Figure 3b-e. We utilized GWAS summary statistics from European, African, East Asian, South Asian, and Hispanic cohorts, along with population-specific LD reference panels from 1000 Genomes Phase 3. Our analysis revealed 1,211 independent genome-wide significant SNPs (defined as r^2^<0.01 in a 1Mb window) in the trans-ancestry T2D GWAS, suggesting that T2D is highly polygenic.

MuGenT has optimal power when there is shared heritability and positive genetic correlation between populations (Figure 3a). Using LD Score regression (LDSC) and alternating pairs of LD reference panels (Bulik-Sullivan et al., 2015), we estimated the genetic correlations of T2D between the five ancestral populations to range from 0.67 to 1.00 (Figure 3b). In our analysis, MuGenT identified 269 genes significantly associated with T2D at the Bonferroni significance level despite lacking any genome-wide significant SNPs. Notably, 45 of these (7.6%; Figure 3c-d) were druggable and not located within 1Mb of any lead SNP from the trans-ancestry T2D GWAS (GWAS p<5x10^−8^, LD r^2^<0.01 in a 1Mb window). Two examples of these newly druggable genes are *FKBP5* (MuGenT p=1.1x10^−9^) and *CDC42BPA* (MuGenT p=1.3x10^−7^ (Figure 3e). While *FKBP5* methylation has been shown to associate with cardiometabolic risk (Ortiz et al., 2018), follow-up gene-based fine-mapping in the *FKBP5* locus (>1.5Mb) identified *TAF11, ANKS1A, TCP11* and *SCUBE3* as the putative drivers (PIP=1.00) and not *FKBP5* (PIP=0). Alternatively, gene-based fine-mapping in the *CDC42BPA* locus (>1.5Mb) identified it as a putative driver, and it is a hypothesized regulator of insulin secretion (Huang et al., 2019).

To further explore population heterogeneity in gene associations with T2D, we employed our MuGenT-PH post-hoc tests across the African, European, East and South Asian, and Hispanic cohorts (Figure 1a). The Spearman rank correlation between gene association p-values from MuGenT and population heterogeneity P-values from MuGenT-PH was 0.37 (p<2.2x10^−16^). MuGenT-PH aggregates SNP-level effect size heterogeneity at the gene level and conducts statistical testing.

Notable examples of between-population heterogeneity in the strength of druggable gene associations with T2D include the *EIF4B* and *KIF11* genes. In the African cohort, the rs374136 SNP in the *EIF4B* gene showed a T2D GWAS association p-value of 2.6x10^−18^, while the next lowest p-value in other populations was 0.09 (observed in Europeans, the largest sample group). The MuGenT-PH p-value for heterogeneity was 1.2x10^−8^. Follow-up tests using our MuGenT-Sel post-hoc test (Figure 1a) indicated an African-specific association of *EIF4B* with T2D, achieving a posterior probability greater than 0.95.

*KIF11* is another druggable gene associated with T2D (MuGenT p=4.5x10^−263^) that exhibited significant population heterogeneity in effect size (MuGenT-PH p=1.2x10^−68^). In the E. Asian cohort (GWAS n=247K), the lead variant rs4933731 had a T2D GWAS p-value of 1.1x10^−26^, but all other populations reported GWAS p-values exceeding 5.5x10^−3^, including the EUR cohort, which was three times larger. Fine-mapping of the *KIF11* locus suggests that the rs7911264 variant is likely causal across all populations except for SAS, with stronger LD patterns observed in EAS and SAS cohorts compared to others (see Supplementary Figure S52). All results from MuGenT and MuGenT-PH analyses for T2D are available in Supplementary Tables S5 and S6.

### xGenT identifies druggable genes in AD

In addition to identifying T2D-associated genes, we evaluated AD-associated risk genes for colocalization and genetic correlation with expression and protein quantitative trait loci (e/pQTL) in brain tissues using our xQTL gene-based association test (xGenT). The results are displayed in Figure 4. The eQTL data were sourced from the cerebellum, cortex, frontal cortex, spinal cord, and hippocampus, while the pQTL data were obtained from cortical tissue.

We show in Methods that in the absence of horizontal pleiotropy the xGenT test statistic is proportional to the total/marginal Mendelian Randomization causal effect sizes of expression in the tested brain tissue on AD risk. Of the 82 genes defining independent loci reported in the largest publicly available AD GWAS to date from Bellenguez et al.^32^, xGenT provides additional evidence of shared eQTL signals for 46 genes (56%) and shared pQTL signals for 32 genes (39%) at the Bonferroni significance level in our tested xQTLs. xGenT results from all gene-based tests using brain eQTLs and pQTLs are available in **Supplementary Tables S7-S8**.

We identified 26 lead druggable genes (e.g., *CHRM5, EGLN1, EZH1, RIOK1, RIPK2, SYK, NTRK1*) with significant Bonferroni-adjusted xGenT p-values in eQTL and pQTL analyses that were located outside 1Mb of any of the 82 AD-associated loci (Figure 4b-c). SNP-level AD and eQTL associations for three of these genes (*RIPK2, NTRK1, RIOK1*) related are displayed in Figure 4c, and Figure 4b demonstrates that gene-level fine-mapping identified *NTRK1* and *RIPK2* as putative drivers with high confidence (PIP=1.00) and *RIOK1* with moderate confidence (PIP=0.58). An example demonstrating the statistical power gained by xGenT through shared disease GWAS and xQTL signals is presented for *RIPK2* in Figure 4d. SNP-level fine-mapping using revealed that each of these three genes had at least one corresponding SNP with a causal PIP greater than 0.90, indicating their status as likely causal AD SNPs.

Using single cell RNA sequencing data from the Religious Orders Study/Memory and Aging Project (ROSMAP; Bennett et al., 2018), we found that all three genes were expressed in glial cells within the cerebral cortex (**Supplementary Figure S54**). This is consistent with existing literature implicating glial cells in AD pathology (Nagele et al., 2004; Uddin et al., 2022). Furthermore, Mendelian Randomization (MR) analyses (*cf.* Methods) of *RIPK2* gene expression in the cerebellum and protein abundance in the cortex suggest a potentially causal role for *RIPK2* in protecting against AD (Figure 4f).

### Multi-omics validated xGenT-predicts druggable genes in AD

**Figure 5:**
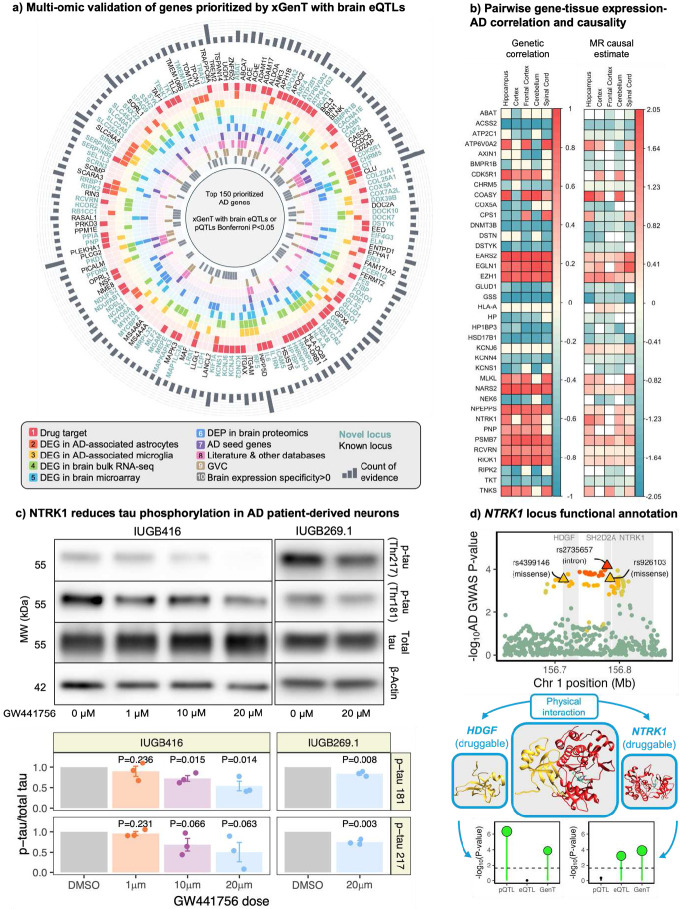
Multi-omics validation of prioritized AD risk genes (a) Multi-omic evidence of association of top 150 of 616 xGenT-prioritized AD risk genes using the sources introduced in Methods. (b) Local genetic correlations between gene expression and AD effects that are marginal, sparse, and calculated using the procedure described in Methods. Mendelian Randomization causal estimates are from univariable analyses and use the procedure described in Methods. Only genes with MR P-values less than 0.05/190 gene-tissue pairs have colored squares. c) AD patient iPSC-derived neurons were treated with *NTRK1* inhibitor GW441756 for 24 hours. Western blot analysis was performed for indicated proteins. Upper: Representative blot. Lower: Quantification of p-tau/total tau for three independent blots. P-values test null hypotheses stating equality between mean p-tau/total tau levels in the DMSO vs each of the other non-DMSO groups. (d) Variants (triangles) and gene locations (grey areas) are annotated and using Ensembl (www.ensembl.org). Points are colored according to the LD with the lead SNP with warmer colors (red, orange) indicating stronger LD and cooler colors (yellow, green) weaker LD. Experimental structures of *HDGF* (left, Protein Data Bank [PDB] ID: 1RI0; Wang et al., 2023, Berman et al., 2000), *NTRK1* (right, PDB ID: 8J5X; Sue et al., 2004), and a predicted complex structure (center) generated using ColabFold (Mirdita et al., 2022). Unstructured and non-interacting regions pruned for clarity. Experimental structures downloaded from the RCSB Protein Data Bank. p-values for ‘eQTL’ are from MuGenT applied to eQTLs for the five brain tissues; P-values for ‘pQTL’ are from GenT applied to pQTLs from the ROSMAP case-control sample (see Methods); p-values for ‘GenT’ are from GenT applied to the AD GWAS summary statistics.

We detected 320 independent genes which had an xGenT Bonferroni p-value less than 0.05 in eQTL or pQTL testing and an AD GenT nominal p-value less than 0.05. These genes had correlations between their GenT test statistics less than 0.01 in a 1Mb window around their base pair midpoints (*cf.* Methods). To validate these prioritized genes, we evaluated supporting multi-omic evidence and displayed the top 150 genes with the strongest supporting data in Figure 5a. Our analysis highlighted several potent drug targets for AD, including *ABAT, ATP2B1, HEXB, CHRM5, KIF1A, GLUD1, KCNS1, RCVRN,* and *RIPK2.* For example, *GLUD1* is upregulated in AD-associated microglia, and its protein (UniProt ID: P00367; The UniProt Consortium, 2023) is elevated in the frontal cortex and hippocampus of the 5xFAD mouse model of AD (Savas et al., 2017). Similarly, *RCVRN* is overexpressed in AD patients compared to controls in bulk RNA-seq data from hippocampal tissue (Magistri et al., 2015; Zhou et al., 2021).

Figure 5b presents marginal genetic correlation and Mendelian Randomization causal effect estimates for each gene expression-brain tissue pair with AD. These estimates suggest that the expression of genes such as *EARS2, EZH1, NARS2, NTRK1, RCVRN,* and *RIOK1* are associated with increased AD risk across multiple brain regions. *NTRK1* consistently demonstrated a strong association with AD risk, as evidence by analyses from GenT, gene-level GenT fine-mapping, xGenT, genetic correlation, and Mendelian Randomization across various brain tissue types with a relatively strong effect in the hippocampus (Figure 5b). *NTRK1* has been shown to associate with other conditions related to aging and cognitive decline such as Parkinson’s disease (Liu et al., 2018), early AD subtype (Cozza et al., 2008), arthritis (Hall et al., 2024), and hippocampal volume and cell survival (de Frutos Lucas et al., 2022; Yang et al., 2023), but literature support for the association between *NTRK1* and AD directly remains limited.

Figure 5d illustrates the distribution of AD GWAS SNP associations within the *NTRK1* locus, highlighting two missense mutations that are marginally associated with AD: rs926103 on the third exon of *SH2D2A* and rs4399146 on the fifth exon of HDGF (Raney et al., 2024). These SNPs serve as pQTLs for *HDGF* (FDR<0.05), but not for *NTRK1* (FDR>0.05) in cortical tissue (Bennett et al., 2018). Gene-based testing of all *cis*-pQTL SNPs in the cortex with GenT, and cis-eQTL SNPs from multiple brain regions with MuGenT, indicated significant pQTLs for *HDGF* but no eQTLs, while *NTRK1* exhibited significant eQTLs without significant pQTLs. Additionally, the *NTRK1* protein physically interacts with *CDK5,* a known AD risk gene associated with tau phosphorylation (Maccioni et al., 2001; Liu et al., 2016).

### Experimental validation of NTRK1 association with AD in iPSC-derived neurons

Given the strong evidence linking *NTRK1* to AD and the limited supporting literature, we pharmacologically targeted *NTRK1* to evaluate its functional connectivity to AD. *NTRK1* plays a crucial role in the development and survival of nerve cells and is also specifically involved with subtyping of sensory neurons (Wehrman et al., 2007). It is primarily expressed in the brain and adrenal glands (Uhlen et al., 2015), and mutations in *NTRK1* are associated with various cancers (Sondka et al., 2018) and pain insensitivity (Indo, 2001).

Our western blot results demonstrated that targeting *NTRK1* reduced the levels of Tau phosphorylation at two key sites (pTau181, p-value=0.008; pTau217, p-value=0.003) in a dose-dependent manner in female patient iPSC-derived neurons (IUGB416) (Figure 5c). Additionally, *NTRK1* targeting significantly decreased Tau phosphorylation at both sites in another patient-derived iPSC neuronal cell line (IUGB269.1). Since hyperphosphorylation of Tau leads to the formation of neurofibrillary tangles, a hallmark AD pathology, our *in vitro* findings suggest that targeting *NTRK1* may help alleviate Tauopathy in AD neurons.

## Discussion

Gene-based association testing offers a powerful alternative to standard SNP-based association methods most commonly used for discovering druggable genes in genome-wide association studies (GWASs). While SNP-based gene discovery faces challenges related to gene assignment and uncontrolled false positive and negative rates, gene-based methods provide greater robustness against these issues. However, existing gene-based tests lack strong theoretical support, leading to inflated error rates.

To address these challenges, we introduced a set of gene-based tests that employ a new inferential approach, effectively controlling false positive and negative rates. This allows for the identification of numerous novel druggable genes associated with complex diseases. We demonstrated the effectiveness of our approach for AD, ALS, MDD, SCZ, and T2D and highlighted select examples of druggable genes with supporting functional evidence.

Through gene-based association testing, we identified 415 potential druggable genes associated with at least one of these diseases. Notable examples include *SYK* for AD and *THRB* for schizophrenia. *SYK* is highly expressed in brain tissue (Sjöstedt et al., 2020) and shows strong genetic correlations with 17 neuropsychiatric and metabolic phenotypes (Supplementary Figure S35). Although the lead SNP in the *SYK* locus (±50Kb) did not reach genome-wide significance (p=2.1x10^−5^; Bellenguez et al., 2022), we found a significant association with AD using both GenT and xGenT, with brain eQTLs (p 2x10^−9^) and pQTLs (p=2.1x10^−9^). *SYK,* primarily expressed in microglia (Zhang et al., 2016; Sjöstedt et al., 2020), works synergistically with *TREM2* in microglia to clear amyloid beta (Wang et al., 2022), the accumulation of which is a key hallmark of AD pathology (Hampel et al., 2021). This study provides the first genome-wide association evidence linking *SYK* to AD, which has previously only been documented in experimental settings.

Additionally, we identified an association between the thyroid hormone receptor gene (*THRB*) and SCZ in a locus that was not detected in previous GWAS. Variants in *THRB* are linked to various phenotypes, including cognitive function (Davies et al., 2015), asthma (Demenais et al., 2017), telomere length (Allaire et al., 2023), myopia (Pickrell et al., 2016), and atrial fibrillation (Sakaue et al., 2021). *THRB* is primarily expressed in cerebral cortical neurons (Sjöstedt et al., 2020) and functions as a thyroid hormone receptor. Functional studies suggest that thyroid hormone is vital for normal brain development (Richard et al., 2023). Given that many SCZ risk factors relate to disruptions in normal brain development (Jones et al., 1994; Murray et al., 2017; Janoutova et al., 2016; Eyles, 2021), we hypothesize that *THRB* may confer SCZ risk by disrupting thyroid signaling pathways during development. Both *THRB* and *SYK* warrant further functional investigation as potential therapeutic targets for SCZ and AD, respectively.

We also applied a multi-ancestry gene-based association test (MuGenT) to T2D across African, European, East Asian, South Asian, and Hispanic populations, identifying 45 druggable genes in loci that were not detected using SNP-level testing in the largest T2D GWAS, which involved over 1.2 million individuals. Our gene-based test demonstrated greater power than trans-ancestry SNP-level GWAS that relies on individual level data, as it reduced the multiple testing burden and did not assume homogenous effect sizes across populations. Notable novel druggable genes include *FKBP5* and *CDC42BPA.* Functional studies have suggested that *FKBP5* expression in subcutaneous adipose tissue may induce insulin resistance (Pereira et al., 2014; Sidibeh et al., 2018), and its methylation could increase the risk of T2D and hyperlipidemia (Ortiz et al., 2018). Additionally, increased activation of the protein encoded by *FKBP5* has been linked to tau aggregation in AD (Agam et al., 2024). *CDC42BPA* has been associated with metabolic traits such as aberrant low-density lipoprotein (Nielsen et al., 2020) and diastolic blood pressure (Warren et al., 2017), as well as smoking initiation (Saunders et al., 2022). Furthermore, it has been implicated in inflammatory responses using CRISPRi screening in human-derived astrocytes (Leng et al., 2022).

We also detected four novel T2D-associated genes using our gene-based test of population heterogeneity: *CA6, CCT3, KRT8,* and *ASIC2.* Previous studies indicate that CA6 protein abundance in plasma differs between T2D patients with and without β-cell dysfunction (Belongie et al., 2017). *CCT3* may be a marker of diabetic nephropathy (Kipp et al., 2021) and *KRT8* has been shown to be involved in the regulation of blood glucose (Alam et al., 2013). *ASIC2,* primarily expressed in neurons, is related to cardiovascular function (Lopez-Ramirez & Gonzalez-Garrido, 2023) and has been linked to metabolic syndrome in mice (Newberry et al., 2024). Collectively, these findings provide epidemiological and biological support for the association between these genes and T2D risk.

Additionally, we highlighted three candidate druggable genes (*RIPK2, NTRK1* and *RIOK1*) associated with AD using gene-level fine-mapping and our gene-based test that integrated brain xQTLs. Although the lead GWAS SNPs for these genes did not achieve genome-wide significance, they exhibited strong xQTL signals, resulting in high significance when tested with xGenT. Each of these genes has previously been validated for association with AD endophenotypes using CRISPRi. For example, CRISPRi testing suggested that *NTRK1* over-expression can cause AD-like neuronal loss (Tian et al., 2019; Gomez-Isla et al., 1997; Mukhin et al., 2017). Additionally, knocking down *RIPK2* in astrocytes is associated with increased inflammatory reactivity, while *RIOK1* knockdown is linked to reduced reactivity (Leng et al., 2022). These associations align with observed genetic correlations between gene expression and AD in the cerebellum (*RIPK2*; Supplementary Figures S16, S33) and hippocampus (*RIOK1*; Supplementary Figures S15, S32), indicating a potential role for brain expression of *RIPK2* and *RIOK1* in influencing neuroinflammation associated with AD (Heneka et al., 2015; Mendes et al., 2015; Kaur et al., 2019).

Our work demonstrates several strengths, including robust statistical properties of our gene-based tests, the discovery of novel empirically supported genes across multiple disease phenotypes, the integration of transcriptomic information, and the availability to apply fine-mapping to gene-based test statistics in large genomic regions. First, our gene-based tests are theoretically grounded and extensively validated through statistical theory and simulations. They require only GWAS summary statistics and an LD reference panel, making them applicable for inter-population gene association inference. Our foundational test, GenT, utilizes a standard gene-based test statistic with a now clearly defined null distribution, overcoming challenges faced by previous methods. The extensions developed from GenT leverage its theoretical foundations, enabling broader and more targeted gene-based inferences than previously available to researchers. We successfully identified novel druggable genes using real-world data across multiple complex diseases, revealing numerous candidate targets that warrant additional experimental investigation. Many of these genes also have historical support from candidate gene or functional studies, strengthening the evidence that our tests can reveal true disease associations that escape detection by traditional SNP-based GWAS methods, a common standard for detecting disease-associated genes. Furthermore, by integrating transcriptomic data in our xGenT tests, we can provide additional insights into the contexts in which specific disease risks arise. Our framework can include any xQTL data, such as those for protein abundance, DNA methylation, gene expression, alternative splicing, and metabolomics.

We acknowledge several potential limitations in our current gene-based framework. Fully leveraging GWAS data from multiple ancestral populations can enhance novel druggable gene discovery only if corresponding population-specific data exists, which is often not the case for many complex diseases. The list of such diseases is extensive, and future research should focus on disseminating all population-specific GWAS and xQTL data, even when sample sizes are small, as these data can be utilized by meta-analytic techniques such as MuGenT. Additionally, optimal assignment of SNPs to genes remains ambiguous in the literature. Consequently, we conservatively assigned SNPs to all genes within 50Kb of the gene body. We show in Supplement Section S12 that 10Kb and 100Kb windows explain approximately the same trait heritability as that which is explained by SNPs in 50Kb windows. Increasing window sizes on average reduce the power of gene-based association testing if they do not capture additional causal SNPs, but increasing SNP set sizes makes transformed gene-based test statistics for fine-mapping converge to their asymptotic distribution faster. Future work should evaluate the extent to which discovery of druggable targets is affected by SNP set window sizes to better detect candidate genes. Finally, while our results provide preliminary evidence of associations between druggable genes and disease phenotypes, further functional analyses are necessary before these genes can be considered safe and effective therapeutic targets.

In conclusion, our study supports the idea that gene-level statistics are more suitable for gene-based inference than traditional SNP-based approaches, enabling researchers to identify a greater number of disease-associated druggable genes. The discrepancies between large scale GWAS and more targeted experimental investigations raise important questions. Many of the genes identified through our enhanced gene-based tests possess strong biological relevance within well-established pathways, suggesting they should be detectable through GWAS. We assert that many genes validated in experimental contexts confer protection or risk for diseases via multiple intricate pathways, making their detection in GWAS inversely proportional to the complexity of these pathways and level of causality involved. Additionally, a disease-associated gene containing multiple causal SNPs is generally more likely to be detected through gene-based association testing than by the lead SNP approach. While our integration of xQTLs in gene-based testing offers insights into biological plausibility for specific disease-gene associations, it is essential to consider simultaneous gene associations with other phenotypes when selecting candidate druggable targets. For example, we show in Supplementary Figure S33 that *RIPK2* expression in cerebellum is negatively correlated with AD, indicating a potentially protective effect, whereas the corresponding genetic correlation with coronary artery disease (CAD) is positive. Therefore, future research must consider that pharmacologically targeting such a gene may reduce risk of one disease while simultaneously increasing the risk of another. The development of methods which can identify these targets is ongoing (e.g., Lorincz-Comi et al., 2025a), though it is still in its early stages.

## Methods

### Data sources for GWAS and drug-target interactions

We used publicly available summary statistics from genome-wide association studies (GWASs) for all gene discovery and estimated ancestry-specific linkage disequilibrium (LD) from the 1000 Genomes Phase 3 (1000 Genomes Project Consortium, 2015) reference panels using PLINKv1.9 (Chang et al., 2015). Disease GWAS summary statistics included those for Alzheimer’s disease (AD; Bellenguez et al., 2022; n=487K EUR), schizophrenia (SCZ; Trubetskoy et al., 2022; n=320K EUR), major depressive disorder (MDD; Howard et al., 2019; n=500K EUR), amyotrophic lateral sclerosis (ALS; Van Rheenen et al., 2021; n=138K EUR), and Type 2 diabetes (T2D; Suzuki et al., 2024; n=752K EUR, n=129K AFR, n=247K EAS, n=42K SAS, n=73K HIS). We also used expression quantitative trait loci (eQTL) summary statistics from GTEx v8 (GTEx Consortium, 2020) for cortex, frontal cortex, hippocampus, cerebellum, and spinal cord tissues in predominantly European populations. All analyses therefore used single nucleotide polymorphisms (SNPs) with minor allele frequency greater than ~0.1%. Our Data Availability statement provides the repositories from which all GWAS summary data used in the main text was downloaded. We defined our list of 3,369 ‘druggable genes’ as those with drug-target interactions by joining lists of interactions from ChEMBL (Gaulton et al., 2017), BindingDB (Gilson et al., 2016), and GtoPdb (Harding et al., 2022) to their mapped molecules in DrugBank (Wishart et al., 2018).

### Single-nuclei RNA-sequencing (snRNA-seq) data

We used individual-level snRNA-seq data from the Religious Orders Study and Rush Memory and Aging Project (ROSMAP; Bennett et al., 2018) to compare gene expression in the prefrontal cortex across the following broad cell type groups: epithelial, fibroblast, glial, immune, neuron, and vascular. Epithelial cells included choroid plexus epithelial cells and ependymal cells; glial cells included astrocytes, microglia, oligodendrocytes, and oligodendrocyte precursor cells; immune cells included choroid plexus macrophages and T-cells; neurons included excitatory and inhibitory neurons; vascular cells included ependymal cells, pericytes, and smooth muscle cells.

### Multi-omics validation data

The results presented in Figure 5a used reference data from multiple sources. These included The Alzheimer’s Cell Atlas (Zhou et al. 2022) for items 1-3 (see Figure 5a key), the Genome-wide Positioning Systems platform for Alzheimer's disease (Zhou et al., 2021) for items 4-7, manual literature searches and verification in the Open Targets Platform (Ochea et al., 2023), Target Illumination GWAS Analytics (Yang et al., 2021), and DisGeNET (Piñero et al., 2016) databases for item 8, the Alzheimer’s Disease Sequencing Project Gene Verification Committee (Greenfest-Allen et al., 2024) for item 9, and GTEx v8 (GTEx Consortium, 2020) for item 10.

### Gene-based association test (GenT)

Our Gene-based association Test (GenT) is a test of association between a gene-specific set of SNPs and a disease phenotype. The primary advantage of GenT over similar existing gene-based tests is that its null distribution is shown to be almost exactly a Gamma distribution with matched first two moments (Covo & Elalouf, 2014; Stewart, 2007) (see Supplement Section S1.1). As a result, it does not rely on complex numerical approximation (Pascal; Lamparter et al., 2016; MAGMA, de Leeuw et al., 2015), simulation (VEGAS; Liu et al., 2010), permutation (MAGMA, LDAK; Berrandou et al., 2023), or an assumed heritability model (LDAK). This makes GenT both theoretically supported and free of the demanding computational burden that repeated sampling imposes. The null distribution of GenT is approximated using a moment method which relies only on unbiased estimation of the LD matrix corresponding to the set of SNPs used for each gene. Let ${\hat{\beta }}^{\ell }={\left({\hat{\beta }}_{1}^{\ell },\ldots ,{\hat{\beta }}_{m^{\ell }}^{\ell }\right)}^{T}$ represent the vector of marginal association estimates corresponding to the ℓth gene and disease phenotype for which there are $m^{\ell }$ SNPs used. GenT tests $H_{0}^{\ell }:\beta^{\ell }=0$ vs $H_{1}^{\ell }:\beta^{\ell }\neq 0$ using the statistic $S_{G}^{\ell }=\sum_{j=1}^{m^{\ell }}T_{j}^{\ell }$ where $T_{j}^{\ell }$ is the standard chi-squared statistic used to test $H_{0j}^{\ell }:\beta_{j}^{\ell }=0$ in GWAS. Under $H_{0j}^{\ell }$, $T_{j}\sim \chi^{2}(1)$ which implies under $H_{0}^{\ell }$ that $S_{G}^{\ell }$ can be very well approximated by Γ(α,ξ) as $m^{\ell }$ grows (Covo & Elalouf, 2014; Stewart, 2007), $E(S_{G}^{\ell }|H_{0}^{\ell })=m^{\ell }$, and $Var(S_{G}^{\ell }|H_{0}^{\ell })=2\text{tr}(R^{\ell }R^{\ell })$ where $R^{\ell }=\text{Corr}({\hat{\beta }}^{\ell })$ is the $m^{\ell }\times m^{\ell }$ matrix of LD correlations between SNPs corresponding to the ℓth gene. This implies $\xi =m^{\ell }∕\text{tr}(2R^{\ell }R^{\ell })$ and $\alpha =m^{\ell }\xi$. We provide additional details about this distributional result and extensive simulations to verify its precision in Supplement Section S1. In practice, we assign all SNPs within 50Kb of the start and end positions of the gene to it as in Liu et al. (2010).

### Fine-mapping multiple genes in a single locus

In standard applications of GenT and the subsequent gene-based tests that we introduce, a single test is performed for each gene. This can lead to inflation of association test statistics across the genome because multiple genes which are physically proximal to each other in local region may share SNPs in LD (Lamparter et al., 2016). Indeed, the empirical evidence suggests that for highly polygenic traits such as T2D, the effect of LD hitchhiking between neighboring gene-specific SNP set can be more severe than that which is encountered in SNP-level analyses. GenT and its extensions are marginal association tests in their original form and do not directly provide a way to infer driver genes in a locus. We introduce three methods to address the issue of inflation from LD and prioritization of driver genes in a locus: (i) post-hoc inflation correction, (ii) prioritization of genes in a locus based on P-value ranking and LD with surround genes, and (iii) fine-mapping of multiple gene-based test statistics in a single locus.

Option (i) is the most straightforward, though its post-hoc adjustment of gene-based P-values beyond that which is due only to high polygenicity may reduce power to detect disease-associated genes. Option (ii) is conceptually equivalent to the clump procedure in PLINK (Chang et al., 2015) and can classify genes into one of three mutually exclusive groups: (a) lead genes, (b) clumped genes, and (c) non-significant genes. Lead genes are significant beyond a user-specified threshold and not correlated with any other genes in the locus beyond a user-defined threshold; clumped genes are significant but are correlated with at least one other gene in the locus which has a smaller gene-based test P-value. Option (iii) uses the limiting distribution of a transformation of the gene-based test statistic $S_{G}^{\ell }=\sum_{j=1}^{m^{\ell }}T_{j}^{\ell }$ from above under the gene-based null hypothesis that $E(S_{G}^{\ell })=m^{\ell }$:

$${\ddot{S}}_{G}^{\ell }≔\frac{1}{\sqrt{2\text{tr}(R^{\ell }R^{\ell })}}(S_{G}^{\ell }-m^{\ell })\overset{D}{\to }N(0,1)\;\;\text{as}\;\;m^{\ell }\to \infty \;.$$

The reason for the transformation of $S_{G}^{\ell }\to {\ddot{S}}_{G}^{\ell }$ is that the joint distribution of multiple gene-based test statistics is unknown, but the limiting distribution of them under the above transformation is multivariate normal. This result states that each transformed gene-based test statistic ${\ddot{S}}_{G}^{\ell }$ follows a standard normal distribution as $m^{\ell }\to \infty$ under $H_{0}^{\ell }$, i.e., as the number of SNPs included in the gene-based association test increases. This property allows us to maximize the penalized joint likelihood for K correlated genes in a single locus using tools such as the Sum of Single Effects (SuSiE) model (Zou et al., 2022) that will perform fine-mapping of the correlated genes. The matrix of correlations between gene-based test statistics is a result of shared LD between their SNP sets and is derived in **Supplement Section S8** for GenT, MuGenT, and xGenT. We provide additional details about fine-mapping of transformed gene-based test statistics in **Supplement Section S9**, and the clumping algorithm of option (ii) described above in **Supplement Section S8**.

### Multiple-ancestry gene-based association test (MuGenT)

The Multiple-ancestry Gene-based association Test (MuGenT) is used to test the association between a gene and multiple traits, which could either be a single disease phenotype in multiple populations, multiple disease phenotypes in a single population, or multiple disease phenotypes in multiple populations. The null hypothesis for this test is that no SNPs in the gene set are associated with any of the tested traits; the alternative hypothesis is that at least one SNP is associated with at least one trait. Figure 3a showed that sharing of causal SNPs across traits/ancestries leads to greater power of the MuGenT test when the effects are genetically correlated, though does not meaningful improve the statistical power of MuGenT when they are not. The rationale for MuGenT and its derivation will be introduced using the context of a single disease phenotype measured in p populations. Let ${\hat{\beta }}_{jk}^{\ell }$ represent the estimate of marginal association between the disease phenotype and jth SNP corresponding to the ℓth gene in the kth population, ${\hat{\beta }}_{k}^{\ell }={\left({\hat{\beta }}_{1k}^{\ell },\ldots ,{\hat{\beta }}_{m^{\ell }k}^{\ell }\right)}^{T}$, and ${\hat{\beta }}_{j}^{\ell }={\left({\hat{\beta }}_{j1}^{\ell },\ldots ,{\hat{\beta }}_{jp}^{\ell }\right)}^{T}$. Also let $R_{k}^{\ell }$ represent the $m^{\ell }\times m^{\ell }$ matrix of LD correlations between SNPs corresponding to the ℓth gene in the kth population. MuGenT tests the null hypothesis $H_{0}^{\ell }:\cap_{k=1}^{p}\beta_{k}^{\ell }=0$ vs $H_{1}^{\ell }:\cup_{k=1}^{p}\beta_{k}^{\ell }\neq 0$ using the statistic $S_{M}^{\ell }=1_{p}^{T}Z_{\ell }^{T}Z_{\ell }1_{p}∕m^{\ell }$ where $Z_{\ell }=(Z_{jk}^{\ell })$ and $Z_{jk}^{\ell }$ is the Z-statistic used to test the association between the jth SNP of the ℓth gene in the kth population and the disease phenotype. Under $H_{0}^{\ell }$, $S_{M}^{\ell }\sim \Gamma (\alpha ;\xi )$ with increasing precision as $m^{\ell }$ grows (Covo & Elalouf, 2014; Stewart, 2007), $E(S_{M}^{\ell }|H_{0}^{\ell })=p$, $Var(S_{M}^{\ell }|H_{0}^{\ell })={\left(m^{\ell }\right)}^{-2}1_{p^{2}}^{T}\gamma^{\ell }1_{p^{2}}$ (see **Supplement Section S3** for the derivation of $\gamma^{\ell }$, which is a transformed sum of all $R_{k}^{\ell }$). This distributional relationship implies that $\xi =p{\left(m^{\ell }\right)}^{2}∕1_{p^{2}}^{T}\gamma^{\ell }1_{p^{2}}$ and α=pξ.

When multiple ancestries are used with MuGenT, the quantity $\gamma^{\ell }≔\text{Cov}[\text{vec}(Z_{\ell }^{T}Z_{\ell })]$, which is the primary component which determines the null distribution of the MuGenT test statistic, has a special patterned structure which is best understood by observing **Supplement Figure S5**. This result shows that LD covariance of the SNP set exists only within populations and not between populations either within or between individual SNPs. This greatly reduces the number of nonzero elements in $\gamma^{\ell }$, and the remaining nonzero covariance elements are summed products of within-population total LD in the SNP set between ancestry pairs. In summary, differing LD patterns between populations are handled by MuGenT since the variance of its test statistic is calculated without assuming LD patterns are the same across populations.

### Multiple-ancestry gene-based test of population heterogeneity (MuGenT-PH)

The Multiple-ancestry Gene-based Test of Population Heterogenity (MuGenT-PH) test is of heterogeneity in gene associations between ancestral populations. Let ${\hat{\beta }}_{jk}^{\ell }$ represent the estimate of marginal association between the disease phenotype and jth SNP corresponding to the ℓth gene in the kth population, ${\hat{\beta }}_{k}^{\ell }={\left({\hat{\beta }}_{1k}^{\ell },\ldots ,{\hat{\beta }}_{m^{\ell }k}^{\ell }\right)}^{T}$, and ${\hat{\beta }}_{j}^{\ell }={\left({\hat{\beta }}_{j1}^{\ell },\ldots ,{\hat{\beta }}_{jp}^{\ell }\right)}^{T}$. The null hypothesis that MuGenT-PH tests is $H_{0}^{\ell }:\cap_{k,s=1;k\neq s}^{p}\beta_{k}^{\ell }=\beta_{s}^{\ell }$ vs $H_{1}^{\ell }:\cup_{k,s=1;k\neq s}^{p}\beta_{k}^{\ell }\neq \beta_{s}^{\ell }$ using the statistic $S_{M-PH}^{\ell }$. The statistic $S_{M-PH}^{\ell }$ is the sum of m SNP-specific statistics $T_{j}^{\ell }$ which are used to test $H_{0j}^{\ell }:\beta_{j}^{\ell }-p^{-1}1_{p}^{T}\beta_{j}^{\ell }=0$ vs $H_{1j}^{\ell }:\beta_{j}^{\ell }-p^{-1}1_{p}^{T}\beta_{j}^{\ell }\neq 0$, which is the null hypothesis of a standard ANOVA test for differences of group means from the grand mean. In this context, $H_{0j}^{\ell }$ tests for population-specific differences in the disease association with SNP j from the population-averaged association. In our estimation of the grand mean in ANOVA, we used an inverse-variance weighting approach in which the kth weight for the jth SNP was equal to $Var{\left({\hat{\beta }}_{jk}\right)}^{-1}$. Under $H_{0j}^{\ell }$, $T_{j}^{\ell }\sim \chi^{2}(p)$, $S_{M-PH}^{\ell }\sim \Gamma (\alpha ,\xi )$ approximately (Covo & Elalouf, 2014; Stewart, 2007), $E(S_{M-PH}^{\ell }|H_{0}^{\ell })=m^{\ell }p$ and $Var(S_{M-PH}^{\ell }|H_{0}^{\ell })≔v^{\ell }$ (see Supplement Section S4 for the derivation of $v^{\ell }$). These results imply that $\xi =m^{\ell }p∕v$ and $\alpha ={\left(m^{\ell }p\right)}^{2}∕v^{\ell }$.

### Multiple-ancestry gene-based test of population pleiotropy (MuGenT-Pleio)

The Multiple-ancestry Gene-based Test of Pleiotropy (MuGenT-Pleio) is used to infer a nonzero association between a gene and disease phenotype in all of p populations. It can also be applied to the study of multiple phenotypes in the same population for an analogous joint inference. Let ${\hat{\beta }}_{jk}^{\ell }$ represent the estimated marginal association between the jth SNP corresponding to the ℓth gene and disease phenotype in the kth population. MuGenT-Pleio simply tests whether any SNP in the set of $m^{\ell }$ corresponding to the ℓth gene is associated with the disease in all populations by applying an intersection-union test (IUT) to each SNP. The null hypothesis of MuGenT-Pleio is $H_{0}^{\ell }:\cap_{j=1}^{m^{\ell }}[\cup_{k=1}^{p}\beta_{jk}^{\ell }\notin {ℛ}_{jk}]$ vs $H_{1}^{\ell }:\cup_{j=1}^{m^{\ell }}[\cap_{k=1}^{p}\beta_{jk}^{\ell }\in {ℛ}_{jk}]$ where ${ℛ}_{jk}$ is the rejection region corresponding to the nominal significance level α used to test each of $H_{0jk}^{\ell }:\beta_{jk}^{\ell }=0$ vs $H_{1jk}^{\ell }:\beta_{jk}^{\ell }\neq 0$. Since $m^{\ell }$ SNPs are considered to correspond to the ℓth gene, and their LD correlation matrix is generally non-diagonal and potentially different between the p populations, we correct the nominal Type I error rate using the following relationship:

(1) $$P\left[\overset{m^{\ell }}{\underset{j=1}{⋃}}\left(\overset{p}{\underset{k=1}{⋂}}\beta_{jk}^{\ell }\in {ℛ}_{jk}\right)|H_{0}^{\ell }\right]\approx 1-{\left(1-\alpha^{p}\right)}^{2m_{eff\cdot }^{\ell }}≔\tilde{\alpha }$$

where $m_{eff}^{\ell }$ is the effective number of independent SNPs in the set of $m^{\ell }$ (Jiang et al., 2022; Galwey et al., 2009; see Supplement Section S5 for the derivation of Equation 1). It can be seen from Equation 1 that the achieved level $\tilde{\alpha }$ can be less or greater than the nominal levels which define ${ℛ}_{jk}$. We correct the nominal level of the standard IUT applied to $m^{\ell }$ SNPs and p populations by expanding or contracting the original rejection regions to form $\tilde{ℛ}$ such that $P[\cup_{j=1}^{m^{\ell }}(\cap_{k=1}^{p}\beta_{jk}^{\ell }\in \tilde{ℛ})|H_{0}^{\ell }]\approx \alpha$. We show in Supplement Section S5 that the quantile of a chi-square distribution defining $\tilde{ℛ}$ which achieves Type I error α is approximately equal to

(2) $$F_{\chi^{2}(1)}^{-1}\left\{1-{\left[1-{\left(1-\alpha \right)}^{\frac{1}{2m_{eff\cdot }^{\ell }}}\right]}^{\frac{1}{p}}\right\},$$

and that this quantile is smaller or larger than the nominal $F_{\chi^{2}(1)}^{-1}(1-\alpha )$ quantile depending on the relationship between p and $m_{eff\cdot }^{\ell }$.

### Multiple-ancestry gene-based test of population-selective effects (MuGenT-Sel)

The Multiple-ancestry Gene-based Test of Selective effects (MuGenT-Sel) test is used to infer that a gene is associated with a trait in a subset of populations or a subset of traits in a single population. We will introduce MuGenT-Sel in the context of two traits in a single population. MuGenT-Sel is developed for inference between two traits and can be applied pairwise to all traits/populations under consideration. Denote ${\hat{\beta }}_{jk}^{\ell }$ as the marginal estimate of association between the jth SNP of $m^{\ell }$ corresponding to the ℓth gene and the kth of 2 traits and denote $Z_{jk}^{\ell }$ as its corresponding Z-statistic used to test $H_{0jk}^{\ell }:\beta_{jk}^{\ell }=0$ in GWAS. Since the goal of MuGenT-Sel is to infer a lack of gene association for one trait only, we develop this method based on the probability of gene associations belonging to one component of a mixed distribution. Note, failure to reject $H_{0k}^{\ell }:\cup_{j=1}^{m^{\ell }}\beta_{jk}^{\ell }=0$ is not sufficient evidence that $H_{0k}^{\ell }$ should be regarded as true with fixed probability given a particular model and so we cannot simply evaluate the rejection and non-rejection of each $H_{0k}^{\ell }$ to infer gene ℓ is only associated with one trait. The null hypothesis of MuGenT-Sel is $H_{0}^{\ell }:(\cap_{k=1}^{2}\beta_{k}^{\ell }\neq 0)\cup (\cap_{k=1}^{2}\beta_{k}^{\ell }=0)$ vs $H_{1}^{\ell }:\beta_{k}^{\ell }=0\cap \beta_{s}^{\ell }\neq 0$ for k≠s, where $\beta_{k}^{\ell }=(\beta_{jk}^{\ell })$. Conceptually, MuGenT-Sel evaluates the probability of $H_{1}^{\ell }$ by comparing the marginal densities of each $\beta_{k}^{\ell }$ in a non-null distribution to the marginal densities in a null distribution.

Where $R_{k}^{\ell }$ is the LD matrix corresponding to the m SNPs assigned to the ℓth gene for the kth trait/population, the ‘null density’ for trait k is $g(z_{k}^{\ell };R_{k}^{\ell })$, where $z_{k}^{\ell }=(Z_{jk}^{\ell })$ and $g(\cdot ;R_{k}^{\ell })$ is the density of a gamma distribution parameterized by $R_{k}^{\ell }$. The ‘non-null’ density for trait k is $g(z_{k}^{\ell };R_{k}^{\ell },\gamma_{k}^{\ell })$, where $\gamma_{k}^{\ell }$ is a mean parameter vector for $z_{k}^{\ell }$ which has undergone LASSO shrinkage and maximizes the marginal penalized multivariate normal likelihood of $z_{k}^{\ell }$. We begin by calculating $p_{k}^{\ell }=g(z_{k}^{\ell };R_{k}^{\ell },\gamma_{k}^{\ell }){\left[g(z_{k}^{\ell };R_{k}^{\ell },\gamma_{k}^{\ell })+g(z_{k}^{\ell };R_{k}^{\ell })\right]}^{-1}$, which is the probability of $z_{k}^{\ell }$ belonging in the non-null distribution given ($R_{k}^{\ell }$, $\gamma_{k}^{\ell }$). Secondly, we transform $p_{k}^{\ell }$ using a shrinkage operator and instead consider $\pi_{k}^{\ell }=d(p_{k}^{\ell })={\left(2p_{k}^{\ell }-1\right)}_{+}$. The purpose of d(⋅) is to transform probabilities of ∕21 to 0, the rationale being that genes which truly belong in their null distribution will only on average be assigned probability ∕21 of belonging in it, but we intend for their probability of belonging in the non-null distribution to be 0 in this case. We then calculate the probability the gene belongs in the non-null distribution for only one trait as $\pi_{1}^{\ell }+\pi_{2}^{\ell }-2\pi_{1}^{\ell }\pi_{2}^{\ell }$ and return it as the MuGenT-Sel result.

### Gene-based test integrating xQTL information (xGenT)

The xQTL Gene-based association Test (xGenT) can be used to infer that SNPs in a gene-specific set share both disease and xQTL signals. xGenT performs this task by including a weighting matrix in the standard GenT test. This weighting matrix is proportional to the xQTL effect sizes, so when none are truly present the xGenT statistic will not be large. When there are both strong xQTL and disease GWAS signals in absolute magnitude for one or more SNPs, the xGenT statistic will be large. Let ${\hat{\beta }}^{\ell }={\left({\hat{\beta }}_{1}^{\ell },\ldots ,{\hat{\beta }}_{m^{\ell }}^{\ell }\right)}^{T}$ and ${\hat{\nu }}_{k}^{\ell }={\left({\hat{\nu }}_{1k}^{\ell },\ldots ,{\hat{\nu }}_{m^{\ell }k}^{\ell }\right)}^{T}$ represent the $m^{\ell }$-length vectors of estimated marginal associations between the m SNPs corresponding to the ℓth gene and the disease phenotype and the kth gene product (e.g., gene expression, protein abundance), respectively. Since there are potentially many gene products and/or tissues which researchers may want to consider as contexts for a potentially causal effect, we let k=1,…,p, define the set 𝒳 to represent them, and combine all xQTL association estimates into the $m^{\ell }\times p$ matrix ${\hat{N}}_{\ell 𝒳}=({\hat{\nu }}_{k})$. Examples may include gene expression in multiple tissues, gene expression and protein abundance in a single tissue, or their combination. Let $\theta_{\ell 𝒳}$ represent the p-length vector of total/marginal causal effects which gene products in the set 𝒳 have on the disease phenotype. The null hypothesis that xGenT tests is $H_{0}^{\ell 𝒳}:N_{\ell 𝒳}\theta_{\ell 𝒳}=0$ vs $H_{1}^{\ell 𝒳}:N_{\ell 𝒳}\theta_{\ell 𝒳}\neq 0$ using the statistic $S_{𝒳}^{\ell }=z_{\ell }^{T}L_{𝒳}^{\ell }z_{\ell }$ where $z_{\ell }[{\hat{\beta }}_{j}^{\ell }∕\text{SE}({\hat{\beta }}_{j}^{\ell })]$, and $L_{𝒳}^{\ell }=\sum_{k=1}^{p}{\hat{\nu }}_{k}^{\ell }{\left({\hat{\nu }}_{k}^{\ell }\right)}^{T}$. It follows that $E(S_{𝒳}^{\ell }|H_{0}^{\ell },L_{𝒳}^{\ell })=m^{\ell }$ and $Var(S_{𝒳}^{\ell }|H_{0}^{\ell },L_{𝒳}^{\ell })=2\text{tr}(L_{𝒳}^{\ell }R^{\ell }L_{𝒳}^{\ell }R^{\ell })$ where $R^{\ell }$ is the $m^{\ell }\times m^{\ell }$ matrix of LD correlations between the $m^{\ell }$ SNPs corresponding to the ℓth gene. This implies that $S_{𝒳}^{\ell }|H_{0}^{\ell }$, $L_{𝒳}^{\ell }∼\Gamma (\alpha ,\xi )$ approximately (Covo & Elalouf, 2014; Stewart, 2007) where $\xi =m^{\ell }∕\mathrm{tr}(2L_{𝒳}^{\ell }R^{\ell }L_{𝒳}^{\ell }R^{\ell })$ and $\alpha =m^{\ell }\xi$. Though the causal effects are neither known nor estimable without performing MR, under the assumption of no horizontal pleiotropy, $\beta_{\ell }=N_{\ell 𝒳}\theta_{\ell 𝒳}$. This implies that under this assumption $S_{𝒳}^{\ell }$ tests total/marginal causality since $E({\hat{\beta }}_{j}^{\ell }{\hat{\nu }}_{jk}^{\ell })=E({\hat{\beta }}_{j}^{\ell })E({\hat{\nu }}_{jk}^{\ell })={\left(\nu_{jk}^{\ell }\right)}^{2}\theta_{\ell k}\Rightarrow E(S_{𝒳G}^{\ell }|H_{0}^{\ell })\propto \theta_{\ell 𝒳}$ (see **Supplementary Section S6** for additional results).

However, like GenT and MuGenT, xGenT is primarily intended to be used as a screening tool and not to evaluate causality. In the previous subsection, we stated that the xGenT test statistic could be proportional to a total/marginal causal effect of (e.g.) gene expression on disease risk only when no horizontal pleiotropy is present. xGenT in its standard form cannot evaluate the extent to which horizontal pleiotropy is present since it is a single-gene screening tool which evaluates association evidence and so should not alone be used to infer causality. A rejected xGenT null hypothesis can generally be interpreted to mean that there is a shared signal between disease SNPs and xQTLs in a gene-specific set, though stricter analytical tools such as MR, transcriptome-wide association study (TWAS), and colocalization should be used if the goal of the analysis is to make a causal inference regarding the relationship between a gene product and disease risk. We demonstrate this sequential testing strategy in Figure 5b, in which we first screened the genome using xGenT then performed follow-up MR analyses on genes with supporting evidence from xGenT. These follow-up analyses provided supporting evidence of potential causality for many genes prioritized by xGenT, but still xGenT alone should not be used to make a causal inference in practice.

### Defining disease-associated druggable genes in novel loci

For T2D, we had access to ancestry-specific and trans-ancestry GWAS summary statistics. To define the set of previously detected T2D-associated genes from existing GWAS, we began by identifying lead SNPs present in the trans-ancestry GWAS. To do this, we used the --clump procedure in PLINKv1.9 five separate times with each of the ancestry-specific LD reference panels from 1000G Phase 3. This provided five sets of lead SNPs in the trans-ancestry T2D GWAS, from which we formed the union set of and regarded all genes within ±1Mb of any of these lead SNPs are previously detected T2D-associated genes. For SCZ, ALS, and MDD, we followed the same procedure but used European LD reference panels. For AD, we started with the complete list of genes identified in Bellenguez et al. (2022) – the largest AD GWAS to date – and regarded the set of previously known AD genes as those within ±1Mb of any genes identified by Bellenguez et al. We used the published list of identified genes because the publicly available set of GWAS summary statistics from Bellenguez et al. contains less genes identified at the level of genome-wide significance than those reported in the published set.

To provide supporting evidence of association for druggable genes we detected in novel loci, we performed fine-mapping of SNPs in each gene-specific set using the sum of single effects (SuSiE) method (Zou et al., 2022). SuSiE intends to construct sets of SNPs which have fixed probability of containing a causal SNP, and we performed it using the susieR package in R (Wang et al., 2020; Zou et al., 2022; R Core Team, 2023). SuSiE used ancestry-specific LD reference panels from 1000 Genomes Phase 3 and included all SNPs within ±100Kb of the midpoint of each gene by base pair position. All raw LD matrices, denoted $R^{\ell }$ for the ℓth gene, were regularized by $R^{\ell }\leftarrow 0.99\times R^{\ell }+0.01\times I^{\ell }$. All SuSiE parameters were fixed at their default values. SuSiE results are interpreted as supporting evidence because the null hypothesis of no association between any SNP in the gene-specific SNP set and the disease – i.e., the null hypothesis which GenT tests – is tested using a completely different form of inference. These results are not interpreted as replications because both SuSiE and gene-based testing use some of the same data.

### Estimating the number of participants needed to detect SYK

We estimated the number of additional participants needed to detect the association between *SYK* and AD in the publicly available AD GWAS summary statistics from Bellenguez et al (2022) in n total participants. We assumed detection of *SYK* by the lead SNP rs10512201, which respectively had estimated effect size and standard error of −0.439 ($\hat{\beta }$) and 0.0103 (s) in the GWAS summary statistics. We assumed $s\propto n^{-1∕2}$ and transformed $\hat{\beta }$ using g accordingly such that $g(\hat{\beta })∼N(g[\beta ],n^{-1})$, defined the genome-wide significance threshold for the statistic $g{\left(\hat{\beta }\right)}^{2}n$ as $29.71\approx Q=F_{\chi_{1}^{2}}^{-1}$ (1-5E-8) and searched a grid of non-centrality parameters $\lambda_{k}={g(\beta )}^{2}n_{k}$ defined by hypothetical sample size $n_{k}$ until $1-F_{\chi_{1,\lambda }^{2}}(Q)$ was equal at least to 0.9, or 90% power to detect the lead *SYK* SNP at the level of genome-wide significance. The R code used to perform this analysis is available at our Github.

### Simulations

We performed simulations to confirm the null distributions of GenT, MuGenT, MuGenT-PH, MuGenT-Pleio, MuGenT-Sel, and xGenT were correctly specified and present most of these results in the **Supplement**. Primary results are presented in the top panels of Figures 2-4. We describe the general procedure for generating simulated data here and present all details of the simulation procedures in the Supplement. All R code used to reproduce our simulation results is available from our Github repository: https://github.com/noahlorinczcomi/gent_analysis. Since all methods are gene-based, we generated GWAS summary statistics which corresponded to a single gene over at least 1,000 replications. We generated all marginal SNP association estimates by transforming their corresponding joint association estimates, which were generated by a multivariate normal distribution with mean vector $b^{\ell }$ and ${\left(nR^{\ell }\right)}^{-1}$ for the ℓth gene, each of which varied in dimension by the specific simulation setup. The vector $b^{\ell }$ is the vector of true joint associations of the SNPs with the trait and was in all cases made to be sparse. Its nonzero elements were selected randomly and, in the case of simulations with MuGenT, were made to be the same across populations. The matrix $R^{\ell }$ was the LD matrix and for many results presented in the main text had a first-order autoregressive structure with correlation parameter equal to 0.9, which we denote as AR1(0.9) in Figures 2-4. This means that two simulated SNPs separated by k base pairs had LD correlation of $0.9^{k}$. We also tested a compound symmetry structure of the simulated LD matrices and present those results in the **Supplement**, which are consistent with all simulation results presented in the main text. In **Supplement Section S13**, we compare the Type I error rate and power of GenT to those of existing methods such as VEGAS (Liu et al., 2010), GATES (Li et al., 2011), ACAT (Liu et al., 2019), and exset (Lorincz-Comi, 2025c). These results demonstrate that on average GenT has a Type I error which is on average better controlled at nominal levels than GATES, VEGAS, and ACAT, and exhibits similar Type I error control to exset across a range of simulation scenarios (Supplement Figure S17). GenT has power to detect gene-level associations that is comparable to exset and VEGAS, which on average was less than the power of GATES and ACAT when power was low. However, we note that these two methods have slightly inflated Type I error and so a comparison of their power to that of GenT, VEGAS, and exset it not fair. On average, GenT completes in 0.1 seconds for a single gene with 500 SNPs.

In the MuGenT simulation results presented in Figure 3, we did not assume all LD matrices to be of the same structure across populations. The parameter n was the GWAS sample size(s). After fixing ($b^{\ell }$, $R^{\ell }$), we generated ${\hat{b}}^{\ell }$, and in Figure 2 ${\hat{R}}^{\ell }$, then the marginal SNP association estimates ${\hat{\beta }}^{\ell }$ as ${\hat{\beta }}^{\ell }=R^{\ell }{\hat{b}}^{\ell }$ and their corresponding variances from $\chi_{n-1}^{2}(n^{2}-n)$ where $\chi_{a}^{2}$ is a central chi-squared distributed variate with a degrees of freedom. This process assumes the genotypes and phenotype are each standardized to have mean 0 and unit variance. Marginal Z-statistics for each SNP were then calculated using the quotient of the estimated marginal effect sizes and their corresponding standard errors. False and true positive rates were determined by calculating the proportion of test results which passed the nominal 5% significance threshold across all simulation replicates. When we estimated the Type I error rate of GenT when the number of individuals in the LD reference panel was finite, we randomly drew ${\hat{R}}^{\ell }$ from a Wishart distribution parameterized by $n_{\text{ref}}$. and $R^{\ell }$, respectively the LD reference panel sample size and matrix of true LD correlations and transformed it to be a correlation matrix.

We restate that GenT and its extensions are marginal gene-based association tests which use SNP sets formed from SNPs in and around each gene body. One test is performed for each gene, and pairs of gene-specific SNP sets may contain SNPs which are in LD with each other. We show in **Supplement Section S8** that this induces correlation between gene-based test statistics which are in the same loci. The effect of shared LD between SNP sets on inflation may be more severe than that which is encountered in SNP-level analyses. Two comments on inflation are worth considering: (i) inflation is not necessarily indicative of inflated Type I error, (ii) for a fixed number of causal genes, the degree of observed genome-wide inflation is proportional to the degree to which genes are localized within gene-dense loci across the genome. Point (i) holds because a gene-based association test is of a marginal null hypotheses, the rejection of which is not incorrect if the gene is non-causal but in LD with a causal gene. Point (ii) In our simulations of Type I error described above, we are evaluating the frequency with which the gene-based null hypothesis for a single gene is incorrectly rejected at a fixed Type I error rate. These simulations are not intended to evaluate the extent to which GenT and its extensions can identify causal genes in a locus. We introduced the gene-based fine-mapping method above and show its simulation performance in **Supplement Section S9**.

### Mendelian Randomization

We performed univariable Mendelian Randomization treating bulk gene expression or protein abundance in select brain tissues as the exposure and AD risk as the outcome and presented the results in Figure 4f and Figure 5b. IVs in each MR analysis were selected as the cis-xQTLs with P-values less than 5 × 10^−8^. We used the vectors of marginal association Z-statistics as inputs to MR as in Lorincz-Comi et al. (2024) and we controlled for LD between eQTL IVs by pre-transforming each Z-statistic vector to remove it. Let ($z_{jk}^{x}$, $z_{jk}^{y}$) represent the tuple of exposure (x) and outcome (y) marginal, untransformed Z-statistic vectors for the jkth exposure-AD pair. We estimated LD between the corresponding $m_{jk}$ SNPs using the 505 European samples in the 1000 Genomes Phase 3 reference panel and denote the estimated matrix as ${\hat{R}}_{jk}$. Pre-transforming ($z_{jk}^{x}$, $z_{jk}^{y}$) to remove LD meant multiplying ($z_{jk}^{x}$, $z_{jk}^{y}$) by F where F∝G and $\mathrm{GG}={\hat{R}}_{jk}^{-1}$. Kollo (2005) proved that $E({\hat{R}}_{jk}^{-1})\approx m_{jk}{\left(505-m_{jk}-1\right)}^{-1}R_{jk}^{-1}=c_{jk}R_{jk}^{-1}$ and so we used $F=\alpha_{jk}G$ to remove LD structure where $\alpha_{jk}=\max(0.5,c_{jk})$. We used the MRBEE method to estimate causal effects, which controls for bias from horizontal pleiotropy and weak instruments (Lorincz-Comi et al., 2024).

### NTRK1 experiments

We targeted *NTRK1* with a selective inhibitor, GW441756, in human iPSC-derived neurons. To generate mature neurons, AD patient iPSCs (IUGB269.1 and IUGB416) from NCRAD (National Centralized Repository for Alzheimer’s Disease) were amplified and maintained in mTeSR medium (STEMCELL Technologies). iPSCs were then induced into neuronal progenitor cells with neural induction medium (STEMCELL Technologies) and continually differentiated into neuronal precursors with forebrain neuron differentiation medium (STEMCELL Technologies). Precursors were matured into a mixed population of excitatory and inhibitory forebrain-type neurons with forebrain neuron maturation medium (STEMCELL Technologies). Patient iPSC-derived neurons were treated with the *NTRK1* inhibitor for 24 hours with the indicated doses (0, 1, 10, and 20 μM in DMSO). Cells were lyzed in N-PER buffer (Thermo Fisher Scientific) supplemented with Protease/Phosphatase Inhibitor Cocktail (Thermo Fisher Scientific) and total protein were collected for Western blot analysis with chemiluminescence system. Primary antibodies for Western blot were as follows: rabbit anti-p-Tau 217 (Cell Signaling Technology, #51625S), 1:1000; rabbit anti-p-Tau 181 (Cell Signaling Technology, #12885S), 1:1000; rabbit anti-Tau (Cell Signaling Technology, #46687S), 1:1000; mouse anti-β-Actin (Proteintech, #66009-1-Ig), 1:1000. Primary antibodies were diluted in PBS/3% bovine serum albumin and incubated overnight at 4 °C. Secondary antibodies were diluted in PBST/5% milk and incubated for 30 minutes at room temperature. Signals were detected using West Pico or West Femto (Thermo Fisher Scientific) ECL substrate. Band intensity was quantified using NIH ImageJ software and used to indicate protein levels.

## Supplementary Material

1

## Data Availability

All code used to generate our simulation and real data analysis results is available at https://github.com/noahlorinczcomi/gent_analysis. We have created an R package for researchers to apply GenT, MuGenT, MuGenT-PH, MuGenT-Pleio, MuGenT-Sel, and xGenT to their own data which is available from https://github.com/noahlorinczcomi/gent, where tutorials are available. We made all summary gene-based association testing and fine-mapping results from analyses from 32 phenotypes available for query and download from https://nlorinczcomi.shinyapps.io/gent.

## Data Availability Statement

We provide gene-based association test results from GenT for AD, ALS, MDD, SCZ in Supplementary Tables 2-4; multi-ancestry gene-based association testing with MuGenT for T2D in Supplementary Table 5; gene-based population heterogeneity testing with MuGenT-PH for T2D in Supplementary Table 6; brain eQTL- and pQTL-integrated gene-based association testing for AD in Supplementary Tables 7 and 8, respectively; and all fine-mapped SNPs for AD, ALS, MDD, SCZ, and all T2D populations in Supplementary Tables 9-17. We created a web application (https://nlorinczcomi.shinyapps.io/gent/) to interactively query and download all gene-based association test and fine-mapping results with GenT and SuSiE for the 32 phenotypes listed in Supplementary Table S18. In our main analyses, GWAS summary statistics for AD were downloaded from https://www.ebi.ac.uk/gwas/studies/GCST90027158; GWAS summary statistics for SCZ were downloaded from https://pgc.unc.edu/for-researchers/download-results/; GWAS summary statistics for ALS were downloaded from https://www.ebi.ac.uk/gwas/studies/GCST90027164; GWAS summary statistics for MDD were downloaded from https://datashare.ed.ac.uk/handle/10283/3203; GWAS summary statistics for T2D were downloaded from https://www.diagram-consortium.org/downloads.html. All other repositories from which we downloaded GWAS summary statistics for phenotypes whose GenT results are presented at https://nlorinczcomi.shinyapps.io/gent/ are available in Supplementary Table S18. GWAS summary statistics for xQTLs in cortex, cerebellum, frontal cortex, hippocampus, and spinal cord tissues were downloaded from the ‘eQTL Tissue-Specific All SNP Gene Associations’ section of https://gtexportal.org/home/downloads/adult-gtex/qtl.
