# Supplementary material for: Combining xQTL and genome-wide association studies from ethnically diverse populations improves druggable gene discovery
Source: Res Sq. 2025 May 28:rs.3.rs-6700169. Preprint. [Version 1] doi: 10.21203/rs.3.rs-6700169/v1 (PMC12154160; doi:10.21203/rs.3.rs-6700169/v1)
Supplement: 1 [file NIHPPrs6700169v1-supplement-1.pdf]

Supplemental information

Tables S1-S4: GenT results for AD, ALS, MDD, and SCZ

Table S5: MuGenT results for Type II diabetes

Table S6: MuGenT-PH (population heterogeneity) results for Type II diabetes

Tables S7-S8: xGenT results for AD (eQTLs, pQTLs)

Tables S9-S12: Fine-mapped SNPs for Alzheimer’s disease, ALS, MDD, and SCZ

Tables S13-S17: Fine-mapped SNPs for Type II diabetes (European, Africa, E. Asian, S. Asian, Hispanic)

Table S18: Repositories from which we downloaded all GWAS summary statistics used in the analysis presented herein and in analogous analyses with an additional 27 phenotypes, the full results of which are available to query and download from our web application.

Tables S19-S23: Gene-level fine-mapping results in disease-associated loci for AD, ALS, MDD, SCZ, and T2D.
